# METTL3-mediated macrophage exosomal NEAT1 contributes to hepatic fibrosis progression through Sp1/TGF-β1/Smad signaling pathway

**DOI:** 10.1038/s41420-022-01036-y

**Published:** 2022-05-19

**Authors:** Bo Shu, Rui-Zhi Zhang, Ying-Xia Zhou, Chao He, Xin Yang

**Affiliations:** 1 Department of General Surgery, The Second Xiangya Hospital, Central South University, Changsha, 410011 Hunan Province PR China; 2 Department of Surgical Operation, The Second Xiangya Hospital, Central South University, Changsha, 410011 Hunan Province PR China

**Keywords:** Diseases, Gastrointestinal diseases

## Abstract

Hepatic fibrosis (HF) is caused by chronic hepatic injury and is characterized by hepatic stellate cells (HSCs) activation. Studies focusing on the function of exosomes derived from macrophages in HF progression are limited. This study aims to identify the roles of exosomal NEAT1 derived from macrophages on HF and the underlying mechanisms. Our studies showed that METTL3 targeted and enhanced NEAT1 expression in macrophages. Exosomal NEAT1 originating from LPS-treated macrophages promoted HSCs proliferation and migration, and induced the expression of fibrotic proteins including collagen I, α-SMA, and fibronectin. Macrophage exosomal NEAT1 contributed to HSCs activation by sponging miR-342. MiR-342 directly targeted Sp1 and suppressed its downstream TGF-β1/Smad signaling pathway, which eventually led to the inhibition of HSCs activation. Depletion of NEAT1 in the macrophage exosomes inhibited HF progression both in vitro and in vivo. Altogether, our study proved that silence of NEAT1 in the macrophage exosomes exerted protective roles against HF through the miR-342/Sp1/TGF-β1/Smad signaling pathway, suggesting a potential therapeutic target in HF treatment.

## Introduction

Hepatic fibrosis (HF) results from chronic damage to the liver where excessive extracellular matrix accumulates [1]. It was reported that lipopolysaccharide (LPS)-treated THP-1 macrophages promoted hepatic stellate cells (HSCs) proliferation and activation and thus led to fibrosis progression [2]. Nevertheless, the mechanisms underlying macrophages regulate HSCs activation and fibrosis have not been fully understood.

N6-methyladenosine (m6A), one of forms of posttranscriptional RNA modification, plays key roles in mRNA stability, splicing, and translation [3]. m6A modulators, including “writers” (METTL3, METTL14, WTAP and RBM15), “easers” (ALKBH5 and FTO), and “readers” (YTHDF1/2/3, HuR and HNRNPA2B1), have been considered to be essential for regulation of cancer biology. m6A catalytic enzyme methyltransferase like 3 (METTL3) was increased following M1 macrophage polarization [4]. Using an online prediction tool (Starbase), we predicted that METTL3 might bind to long non-coding RNA (lncRNA) NEAT1 [5]. NEAT1 promoted LPS-induced inflammatory response in macrophages [6, 7]. Furthermore, NEAT1 was reported to accelerate the progression of HF [8–10]. Nevertheless, the relationship between METTL3 and NEAT1 as well as whether exosomal NEAT1 plays a role in the crosstalk between HSCs and macrophages during HF need to be further explored.

MiRNA-342 (miR-342) was reported to inhibit the progress of renal interstitial fibrosis [11]. MiR-342 was also documented to be a potent suppressor in hepatocellular carcinoma [12]. NEAT1 may direct target miR-342 as predicted by the online prediction tool (Starbase). However, whether miR-342 is involved in regulating HF remains to be further elucidated. The activation of the Sp1/TGF-β1/Smad pathway was associated with the progress of fibrogenesis of many organs, including atrial fibrosis, liver fibrosis, and endometrial fibrosis [13–15]. Our bioinformatics analysis reveals that miR-342 harbors predictive binding sites for Sp1 mRNA. But, the exact role of the miR-342 on the regulation of the Sp1/TGF-β1/Smad signaling pathway in HF is not clear.

In the present study, we aimed to investigate the functional role and underlying mechanism of METTL3-medicated macrophage exosomal NEAT1 in HF. We speculated that NEAT1 in exosomes secreted from LPS-activated macrophages suppressed miR-342 expression, which further activated the Sp1/TGF-β1/Smad signaling pathway and caused activation of HSCs, eventually led to accelerated progress of HF.

## Results

### NEAT1 expression is increased in exosomes derived from LPS-treated THP-1 macrophages

**Fig. 1:**
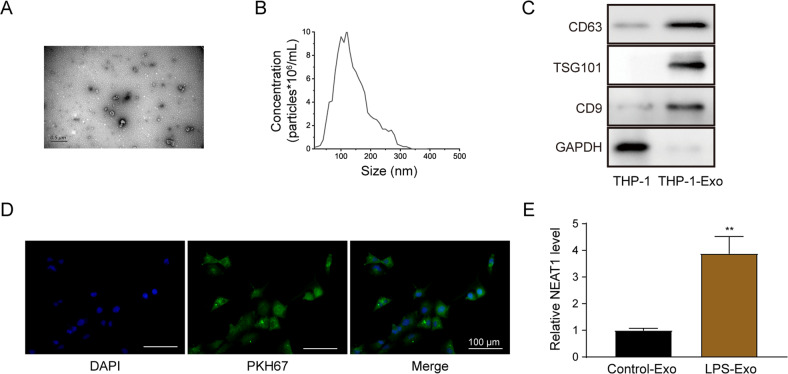
NEAT1 expression is increased in exosomes derived from LPS-treated THP-1 macrophages. **A**, **B** Characterization of exosomes isolated from THP-1 macrophages using TEM (**A**) and NTA (**B**) analyses. **C** Western blot analysis of the expression levels of CD63, TS101, and CD9 in exosomes isolated from THP-1 macrophages. **D** The uptake of PKH67-labeled exosomes by LX-2 cells. Scale bar: 100 μm. **E** qRT-PCR detection of the NEAT1 expression in exosomes isolated from LPS-treated THP-1 macrophages and non-treated macrophages. **p < 0.01.

The exosomes from THP-1 macrophages were isolated and characterized using transmission electron microscope (TEM) and nanoparticle tracking analyzing (NTA) analyses (Fig. 1A, B). The exosomes showed typical rounded with an average diameter of 100–150 nm. The expression of specific exosomal markers CD9, CD63, and TSG101 was further quantified (Fig. 1C). As shown in Fig. 1D, the exosomes marked by PKH67 were situated in the cytoplasm of LX-2 cells. LX-2 cells incubated with exosomes from LPS-treated THP-1 macrophages showed a significantly higher NEAT1 expression (Fig. 1E). Taken together, NEAT1 expression was induced in exosomes derived from LPS-treated THP-1 macrophages.

### Exosomes extracted from LPS-treated THP-1 macrophages enhance the activation of HSCs

**Fig. 2:**
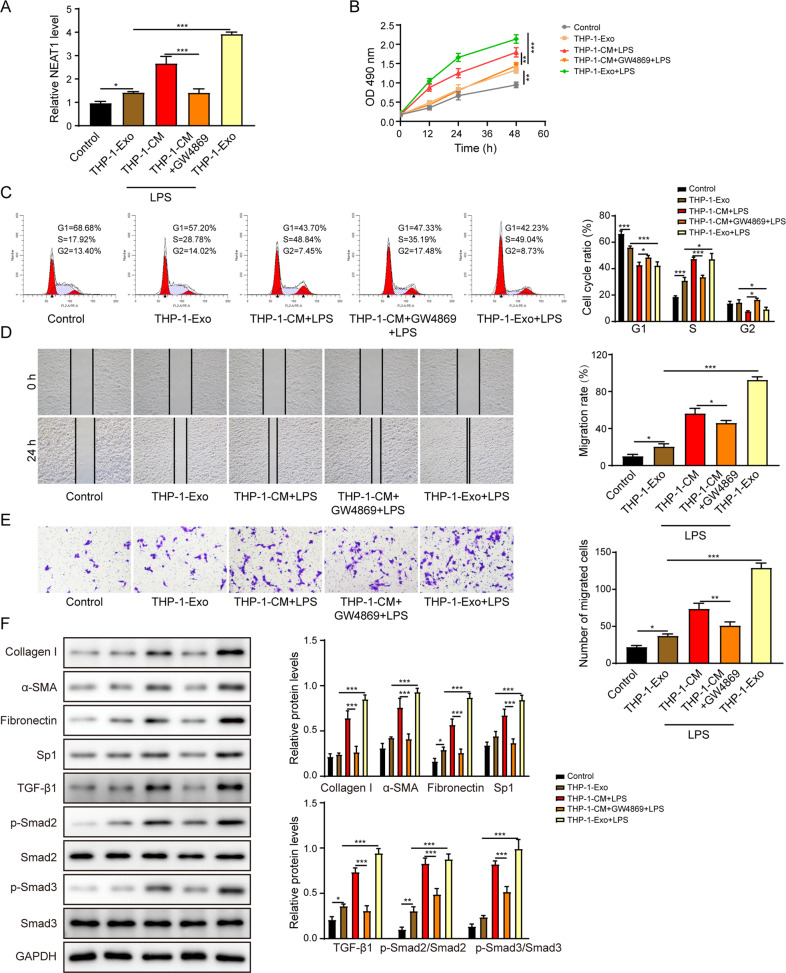
Exosomes extracted from LPS-treated THP-1 macrophages enhance the activation of HSCs. **A** The NEAT1 expression level was determined by qRT-PCR. **B** The proliferation of the LX-2 cells was examined by the CCK-8 assay. **C** The cell cycle of the LX-2 was examined by the flow cytometry. **D**, **E** The migration of LX-2 was determined by the scratch assay (**D**) and the transwell assay (**E**). **F** Western blot analysis of the protein levels of collagen I, α-SMA, fibronectin, Sp1, TGF-β1, p-Smad2, Smad2, p-Smad3, and Smad3. *p < 0.05; **p < 0.01; ***p < 0.001.

As indicated by Fig. 2A, the NEAT1 expression was significantly increased in LX-2 cells that were treated with conditional medium (CM) or exosomes derived from LPS-treated THP-1 macrophages. However, the treatment of GW4869 (an inhibitor of exosome generation) reversed the stimulation effect of CM. Similarly, the cell proliferation (Fig. 2B), cell cycle transition from G1 phase to S phase (Fig. 2C), and migration (Fig. 2D, E) of LX-2 cells were enhanced following the treatment of CM or exosomes, whereas the treatment of GW4856 reversed the influences of CM on LX-2 cells (Fig. 2B–E). Treatment of LX-2 cells with CM or exosomes largely increased the levels of collagen I, α-SMA and fibronectin (Fig. 2F). Similarly, Sp1, TGF-β1, phosphorylated-Smad2 and phosphorylated-Smad3 levels were also elevated in LX-2 cells after treatment with CM or exosomes. Nevertheless, GW4869 reversed the effects of CM treatment (Fig. 2F). Altogether, these data suggest that exosomes secreted from LPS-treated THP-1 macrophages contributed to the activation of HSCs via Sp1/TGF-β1/Smad signaling pathway.

### Exosomal NEAT1 from THP-1 macrophages promotes the activation of HSCs

**Fig. 3:**
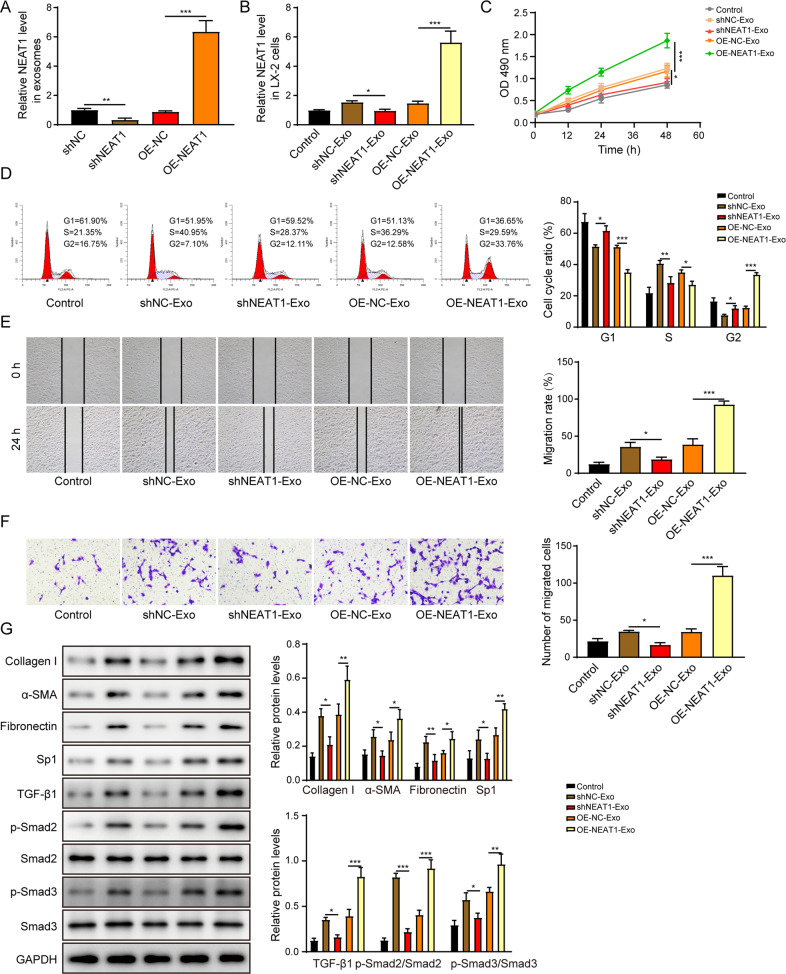
Exosomal NEAT1 from THP-1 macrophages promotes the activation of HSCs. **A** The NEAT1 expression level was determined by qRT-PCR in exosomes from THP-1 macrophages. **B** The NEAT1 expression level was determined by qRT-PCR in LX-2 cells. **C** The proliferation of the LX-2 was examined by the CCK-8 assay. **D** The cell cycle of the LX-2 was examined by the flow cytometry. **E**, **F** The migration of LX-2 was determined by the scratch assay (**E**) and the transwell assay (**F**). **G** The protein levels of collagen I, α-SMA, fibronectin, Sp1, TGF-β1, p-Smad2, Smad2, p-Smad3, and Smad3 were measured by western blot. *p < 0.05; **p < 0.01; ***p < 0.001.

To identify the function of exosomal NEAT1 from THP-1 macrophages on HSCs, we knocked down or overexpressed NEAT1. The success of transfection was confirmed by determining the NEAT1 expression in exosomes derived from indicated THP-1 macrophages (Fig. 3A). NEAT1 expression was significantly decreased or increased following the treatment of NEAT1-depleted or -overexpressed exosomes in LX-2 cells respectively (Fig. 3B). Incubation with NEAT1-depleted exosomes inhibited the cell proliferation (Fig. 3C), cell cycle transition (Fig. 3D), and migration (Fig. 3E, F). Furthermore, treatment with NEAT1 overexpressed exosomes promoted the expression levels of collagen I, α-SMA, fibronectin, Sp1, TGF-β1, phosphorylated-Smad2 and phosphorylated-Smad3 in LX-2 cells (Fig. 3G). However, treatment with NEAT1-depleted exosomes exerted the opposite effects (Fig. 3C–G). Collectively, exosomal NEAT1 from THP-1 macrophages enhanced HSCs activation via activation of Sp1/TGF-β1/Smad signaling pathway.

### METTL3 facilitates NEAT1 expression in THP-1 macrophages

**Fig. 4:**
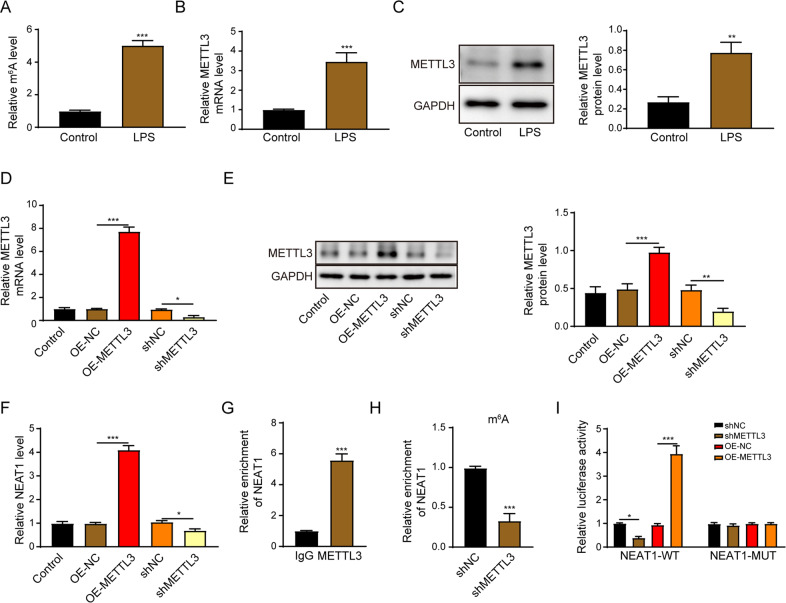
METTL3 facilitates NEAT1 expression in THP-1 macrophages. **A** The m6A expression level in THP-1 macrophages or LPS-activated THP-1 macrophages. **B**, **C** The METTL3 mRNA and protein levels were determined by qRT-PCR (**B**) and western blot (**C**) in THP-1 macrophages or LPS-activated THP-1 macrophages. **D**, **E** The METTL3 mRNA and protein levels were determined by qRT-PCR (**D**) and western blot (**E**) in METTL3 overexpressed or depleted THP-1 macrophages. **F** The NEAT1 expression level was determined by qRT-PCR in METTL3 overexpressed or depleted THP-1 macrophages. **G** METTL3 RIP-qPCR analysis of NEAT1 enrichment in THP-1 macrophages. **H** m6A RIP-qPCR analysis of NEAT1 enrichment in shMETTL3-transfected THP-1 macrophages. **I** The direct interaction between METTL3 and NEAT1 was verified by luciferase reporter assay. *p < 0.05; **p < 0.01; ***p < 0.001.

The m6A level was significantly upregulated in LPS-activated THP-1 macrophages (Fig. 4A). The mRNA and protein levels of METTL3 were significantly enhanced under this treatment (Fig. 4B, C). Moreover, the transfection efficiency of METTL3 was proved by Fig. 4D, E. METTL3 overexpression significantly enhanced the NEAT1 expression. In contrast, silence of METTL3 led to the downregulation of NEAT1 (Fig. 4F). NEAT1 was enriched by the METTL3 antibody (Fig. 4G). m6A RIP analysis illustrated that the knockdown of METTL3 suppressed the enrichment of NEAT1 with m6A (Fig. 4H). Additionally, METTL3 overexpression led to a significant increase of luciferase activity in wild type of NEAT1 (NEAT1-WT) reporter, whereas it had no impact in mutant of NEAT1 (NEAT1-MUT) reporter group (Fig. 4I).

### METTL3 promotes NEAT1 expression in Kupffer cells and NEAT1 expression is also increased in exosomes derived from LPS-treated Kupffer cells

We then isolated Kupffer cells from mouse liver and followed by overexpressing or silencing METTL3 expression (Supplementary Fig. 1A, B). METTL3 overexpression significantly increased NEAT1 expression. In contrast, downregulation of METTL3 had the opposite effect on NEAT1 expression (Supplementary Fig. 1C). NEAT1 was enriched by the METTL3 antibody in Kupffer cells (Supplementary Fig. 1D). These results indicated that METTL3 also promoted NEAT1 expression in Kupffer cells.

Exosomes from Kupffer cells were characterized by TEM (Supplementary Fig. 1E), NTA (Supplementary Fig. 1F) and western blot (Supplementary Fig. 1G). The exosomes showed a typical rounded structure with an average diameter of around 100–150 nm and the expression of specific exosomal markers CD9, CD63, and TSG101 were also identified. As indicated by Supplementary Fig. 1H, a significantly increased NEAT1 expression was observed in exosomes isolated from LPS-treated Kupffer cells.

### Exosomes extracted from LPS-treated Kupffer cells enhance the activation of primary HSCs

As indicated by Supplementary Fig. 2A, the NEAT1 expression was significantly enhanced in primary HSCs treated with exosomes derived from LPS-induced Kupffer cells. Similarly, the cell proliferation (Supplementary Fig. 2B), and migration (Supplementary Fig. 2C, D) of primary HSCs were increased following the treatment of exosomes derived from LPS-treated Kupffer cells. Treatment of primary HSCs with exosomes derived from LPS-activated Kupffer cells also increased the expression levels of collagen I, α-SMA, fibronectin, Sp1, TGF-β1, phosphorylated-Smad2 and phosphorylated-Smad3 (Supplementary Fig. 2E). These results indicated exosomal NEAT1 extracted from Kupffer cells also enhanced the activation of primary HSCs.

### NEAT1 promotes HSCs activation via negatively regulating miR-342

**Fig. 5:**
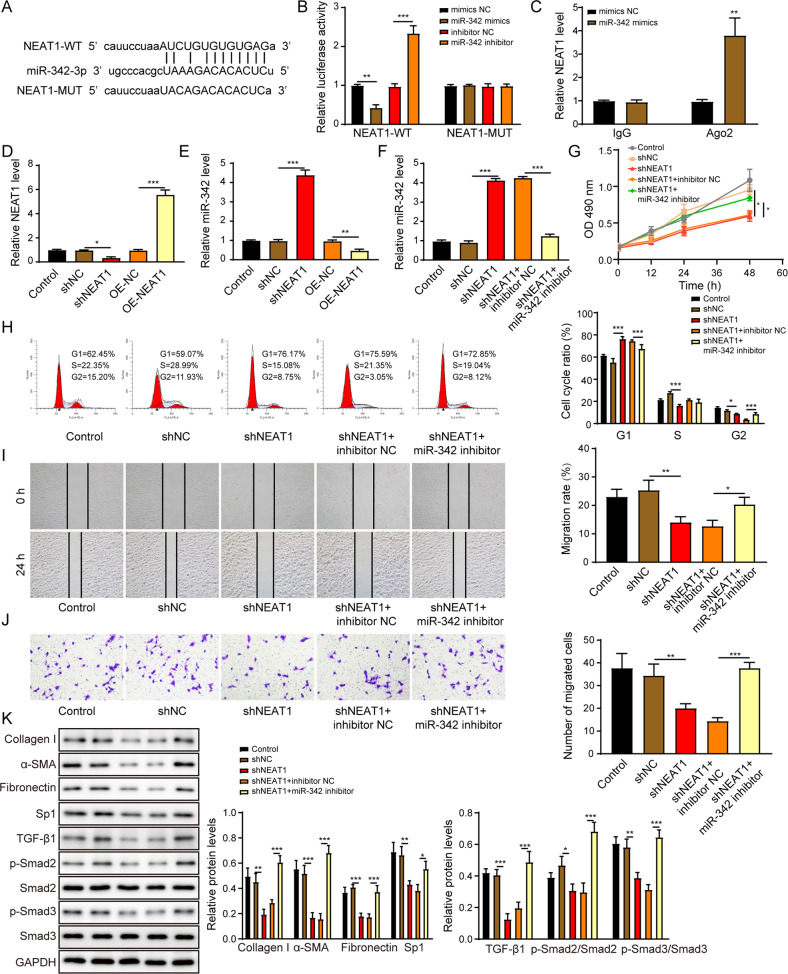
NEAT1 promotes HSCs activation via negatively regulating miR-342. **A** Schematic diagram of the miR-342 binding sites on NEAT1. **B** The dual-luciferase activity of the NEAT1 wild-type sequence (NEAT1-WT) and NEAT1 mutant sequence (NEAT1-MUT) in LX-2 cells transfected with miR-342 mimics or miR-342 inhibitor. **C** Relative NEAT1 expression level in Ago2 immunoprecipitates compared to IgG immunoprecipitates. **D** NEAT1 expression level was overexpressed or knocked down in LX-2 cells by qRT-PCR assay. **E** The impact of NEAT1 knockdown or overexpression on miR-342 expression in LX-2 cells was measured by qRT-PCR. **F** The impact of NEAT1 and miR-342 knockdown on miR-342 expression in LX-2 cells was measured by qRT-PCR. **G** The LX-2 cell proliferation was examined by the CCK-8 assay. **H** The cell cycle of the LX-2 was examined by the flow cytometry. **I**, **J** The migration of LX-2 was determined by the scratch assay (**I**) and the transwell assay (**J**). **K** The protein levels of collagen I, α-SMA, fibronectin, Sp1, TGF-β1, p-Smad2, Smad2, p-Smad3, and Smad3 were measured by western blot. *p < 0.05; **p < 0.01; ***p < 0.001.

It was revealed by the online prediction tool (http://starbase.sysu.edu.cn/) that NEAT1 potentially bound to miR-342 (Fig. 5A). MiR-342 mimics significantly reduced the luciferase activity of LX-2 cells transfected with NEAT1-WT but not transfected with NEAT1-MUT (Fig. 5B). As indicated by Fig. 5C, NEAT1 was enriched by the Argonaute 2 (Ago2) antibody in miR-342 overexpressed LX-2 cells. We then overexpressed or knocked down NEAT1 expression in LX-2 cells and the success of the transfection was demonstrated in Fig. 5D. NEAT1 overexpression significantly reduced the expression of miR-342, whereas silence of NEAT1 enhanced the miR-342 level (Fig. 5E). The promotion effect of NEAT1 depletion on miR-342 expression in LX-2 cells was reversed after transfection with miR-342 inhibitor (Fig. 5F). The miR-342 expression was also significantly decreased in primary HSCs treated with exosomes derived from LPS-induced Kupffer cells (Supplementary Fig. 2F).

As illustrated in Fig. 5G–J, silence of NEAT1 suppressed the cell proliferation (Fig. 5G), cell cycle transition (Fig. 5H), and migration (Fig. 5I, J) of LX-2 cells, while miR-342 depletion could rescue these effects. More importantly, the knockdown of miR-342 attenuated the inhibitory effects of NEAT1 depletion on pro-fibrotic markers, including collagen I, α-SMA, fibronectin, and Sp1/TGF-β1/Smad signaling pathway in LX-2 cells (Fig. 5K). In summary, NEAT1 activated HSCs activation by suppressing miR-342 expression.

### NEAT1 overexpressed exosomes enhance the activation of HSCs by inhibiting miR-342

**Fig. 6:**
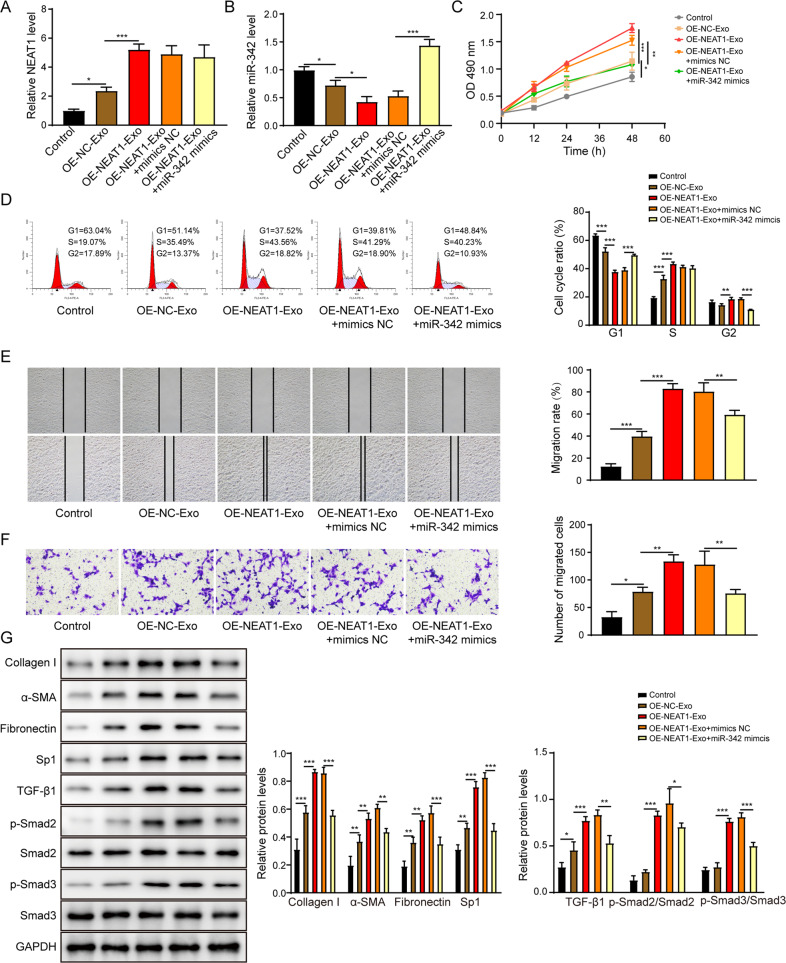
NEAT1 overexpressed exosomes enhance the activation of HSCs by inhibiting miR-342. **A**, **B** The NEAT1 (**A**) and miR-342 (**B**) expression levels in LX-2 cells were assessed via qRT-PCR. **C** The LX-2 cell proliferation was examined by the CCK-8 assay. **D** The cell cycle of the LX-2 was examined by the flow cytometry. **E**, **F** The migration of LX-2 was determined by the scratch assay (**E**) and the transwell assay (**F**). **G** The protein levels of collagen I, α-SMA, fibronectin, Sp1, TGF-β1, p-Smad2, Smad2, p-Smad3 and Smad3 were measured by western blot. *p < 0.05; **p < 0.01; ***p < 0.001.

After co-cultured with exosomes extracted from NEAT1 overexpressed THP-1 macrophages, LX-2 cells displayed significantly increased NEAT1 expression but decreased miR-342 expression (Fig. 6A, B). Nevertheless, transfection with miR-342 mimics reversed the exosomes-induced the inhibition of miR-342 expression in LX-2 cells (Fig. 6B). LX-2 cells incubated with NEAT1 overexpressed exosomes showed enhanced proliferation (Fig. 6C), cell cycle transition (Fig. 6D), and migration (Fig. 6E, F). But miR-342 overexpression attenuated exosomes-induced effects on function of LX-2 cells. As illustrated in Fig. 6G, treatment with NEAT1 overexpressed exosomes promoted collagen I, α-SMA, fibronectin, Sp1, TGF-β1, phosphorylated-Smad2 and phosphorylated-Smad3 protein levels in LX-2 cells, whereas miR-342 overexpression exerted the opposite effects. Taken together, NEAT1 overexpressed exosomes facilitated the HSCs activation by inhibiting miR-342.

### MiR-342 suppresses HSCs activation by negatively mediating Sp1

**Fig. 7:**
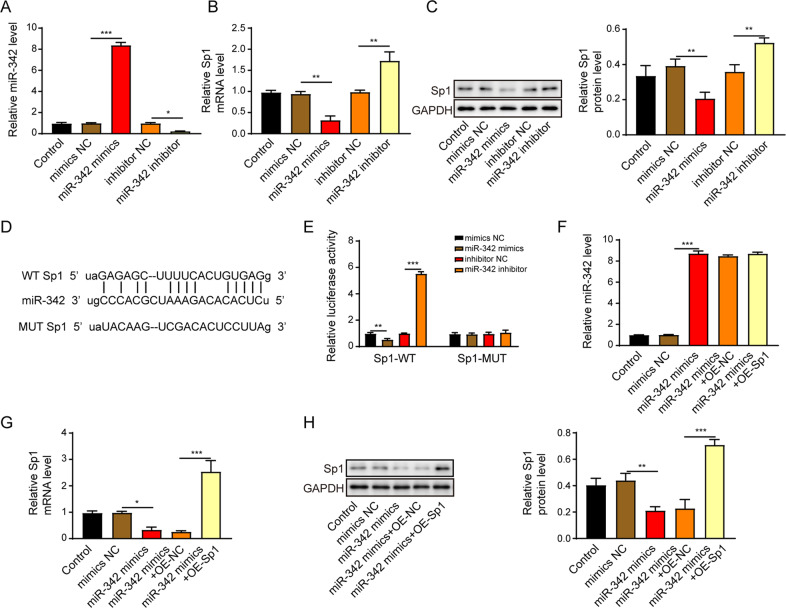
MiR-342 directly suppresses Sp1 expression in LX-2 cells. **A** The miR-342 expression level in LX-2 cells was assessed via qRT-PCR. **B**, **C** Sp1 mRNA (**B**) and protein (**C**) levels were determined using qRT-PCR and western blot, respectively. **D** Schematic diagram of the miR-342 binding sites on Sp1. **E** The dual-luciferase activity of the Sp1-WT and Sp1-MUT in LX-2 cells transfected with miR-342 mimics or miR-342 inhibitor. **F** The miR-342 expression level in LX-2 cells was assessed via qRT-PCR. **G**, **H** Sp1 mRNA (**G**) and protein (**H**) levels were determined using qRT-PCR and western blot, respectively. *p < 0.05; **p < 0.01; ***p < 0.001.

**Fig. 8:**
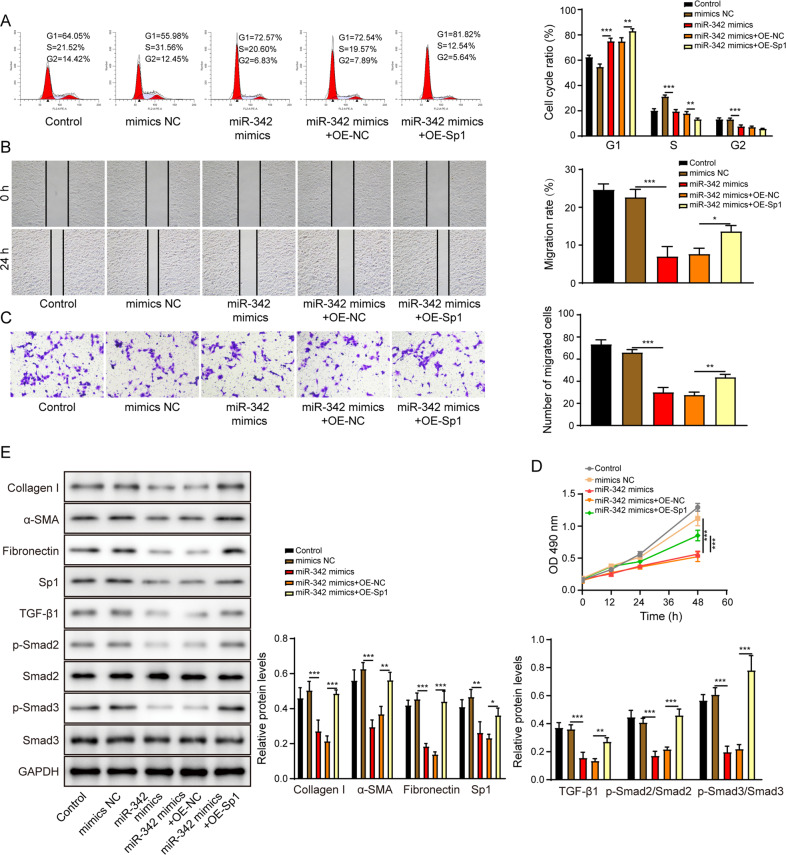
MiR-342 suppresses HSCs activation by negatively mediating Sp1. **A** The cell cycle of the LX-2 was examined by the flow cytometry. **B**, **C** The migration of LX-2 was determined by the scratch assay (**B**) and the transwell assay (**C**). **D** The LX-2 cell proliferation was examined by the CCK-8 assay. **E** The protein levels of collagen I, α-SMA, fibronectin, Sp1, TGF-β1, p-Smad2, Smad2, p-Smad3, and Smad3 were measured by western blot. *p < 0.05; **p < 0.01; ***p < 0.001.

To elucidate the molecular mechanism of miR-342 in regulating HSCs activation, we investigated the interaction between miR-342 and Sp1. We transfected the LX-2 cells with miR-342 mimics or inhibitor, and the efficacy of transfection was displayed in Fig. 7A. The expression of Sp1 was upregulated in LX-2 transfected with miR-342 inhibitor but downregulated in miR-342 mimics group (Fig. 7B, C). The bioinformatics analysis tool (http://starbase.sysu.edu.cn/) predicted that miR-342 potentially bound to 3′-untranslated region (UTR) of Sp1 (Fig. 7D). MiR-342 mimics resulted in a decreased luciferase activity of wild type of Sp1 (Sp1-WT). Moreover, the miR-342 inhibitor led to increased luciferase activity of mutant of Sp1 (Sp1-WT) (Fig. 7E). Nevertheless, the luciferase activity of Sp1-MUT was not affected by miR-342 mimics or inhibitor, indicating miR-342 directly targeted Sp1 (Fig. 7E). MiR-342 overexpression dramatically induced miR-342 expression and suppressed Sp1 mRNA and protein levels, but Sp1 overexpression had no effects on miR-342 (Fig. 7F) and reversed the Sp1 expression (Fig. 7G, H). The cell cycle transition (Fig. 8A), migration (Fig. 8B, C), and proliferation (Fig. 8D) of LX-2 cells were inhibited by miR-342 mimics but were promoted by the overexpression of Sp1. More importantly, overexpression of Sp1 significantly reversed the miR-342 mimics-induced inhibition of pro-fibrotic markers, including collagen I, α-SMA, fibronectin, and TGF-β1/Smad signaling (Fig. 8E). Altogether, miR-342 inhibited HSCs activation by suppressing Sp1/TGF-β1/Smad signaling.

### Exosomal NEAT1 from THP-1 macrophages facilitates HSCs activation via mediating Sp1

**Fig. 9:**
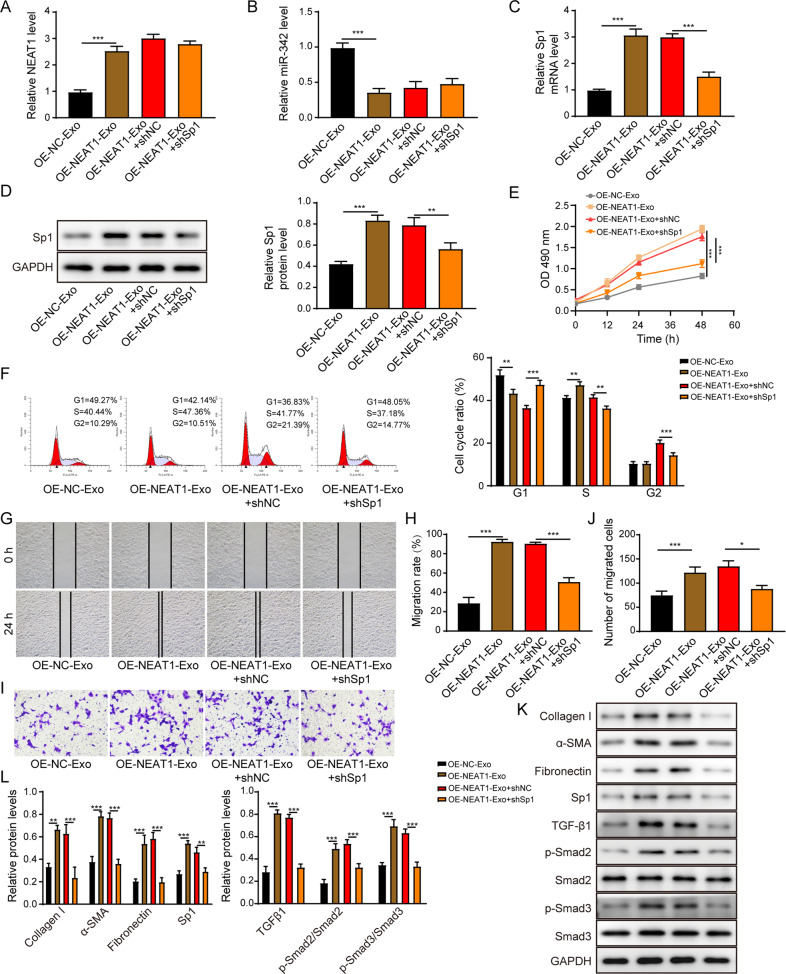
Exosomal NEAT1 from THP-1 macrophages facilitates HSCs activation via mediating Sp1. **A**, **B** The NEAT1 (**A**) and miR-342 (**B**) expression levels in LX-2 cells were assessed via qRT-PCR. **C**, **D** Sp1 mRNA (**C**) and protein (**D**) levels were determined using qRT-PCR and western blot, respectively. **E** The LX-2 cell proliferation was examined by the CCK-8 assay. **F** The cell cycle of the LX-2 was examined by the flow cytometry. **G**–**J** The migration of LX-2 was determined by the scratch assay (**G**, **H**) and the transwell assay (**I**, **J**). **K**, **L** The protein levels of collagen I, α-SMA, fibronectin, Sp1, TGF-β1, p-Smad2, Smad2, p-Smad3 and Smad3 were measured by western blot. *p < 0.05; **p < 0.01; ***p < 0.001.

Treatment with NEAT1 overexpressed exosomes significantly increased NEAT1 and Sp1 expression levels but decreased miR-342 level in LX-2 cells. Silence of Sp1 did not influence these effects on NEAT1 and miR-342 levels but led to the opposite effect on Sp1 expression (Fig. 9A–D). The proliferation (Fig. 9E), cell cycle transition (Fig. 9F), and migration (Fig. 9G–J) of LX-2 cells were promoted following co-culture with NEAT1 overexpressed exosomes but inhibited by Sp1 depletion. NEAT1 overexpressed exosomes enhanced the expression levels of pro-fibrotic factors, including collagen I, α-SMA, fibronectin, and Sp1/TGF-β1/Smad signaling pathway activation. However, these promoting effects were reversed following shSp1 transfection in LX-2 cells (Fig. 9K, L). Collectively, NEAT1 in THP-1 macrophage exosomes facilitated HSCs activation via mediating Sp1.

### Exosomal NEAT1 contributes to HF progression in rat

**Fig. 10:**
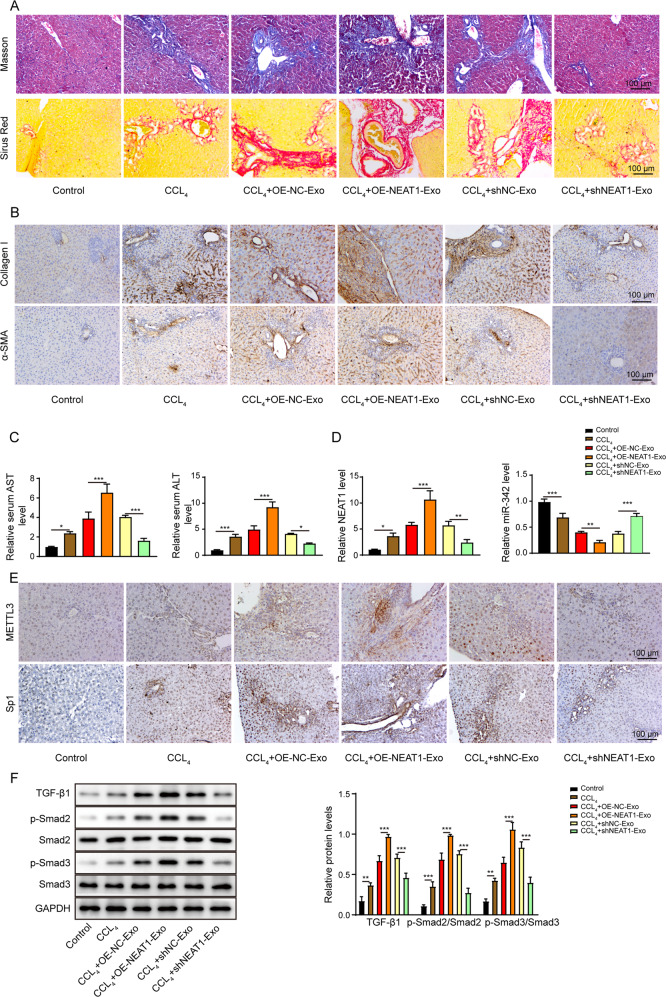
Exosomal NEAT1 contributes to HF progression in rat. **A** Representative images of Masson and Sirus Red stainings in liver tissues from Sprague-Dawley rats. Scale bar: 100 μm. **B** Representative images of IHC staining of pro-fibrotic factors collagen I and α-SMA in liver tissues from Sprague-Dawley rats. Scale bar: 100 μm. **C** ELISA detected serum AST and ALT levels from Sprague-Dawley rats. **D** qRT-PCR detection of NEAT1 and miR-342 expression levels in liver tissues from Sprague-Dawley rats. **E** Representative images of IHC staining of METTL3 and Sp1 in liver tissues from Sprague-Dawley rats. Scale bar: 100 μm. **F** Western blot results of the expression levels of TGF-β1, p-Smad2, Smad2, p-Smad3, and Smad3 in liver tissues from Sprague-Dawley rats. *p < 0.05; **p < 0.01; ***p < 0.001.

The rat in the CCL_4_-induced group displayed elevated liver fibrosis level, as evidenced by accumulated collagen in livers by Masson and Sirus Red stainings (Fig. 10A). Moreover, the rat in the CCL_4_ group showed significantly higher levels of alanine aminotransferase (ALT) and aspartate aminotransferase (AST) in serum as well as increased protein level of collagen I and α-SMA (Fig. 10B, C). Importantly, injection of NEAT1-depleted exosomes attenuated the CCL_4_-induced liver fibrosis and mitigated the AST and ALT levels as well as pro-fibrotic makers, including collagen I and α-SMA. Liver fibrosis level in rat was further elevated following the injection of NEAT1 overexpressed exosomes (Fig. 10A–C). Furthermore, the CCL_4_ group also showed the remarkably enhanced NEAT1 (Fig. 10D), METTL3, Sp1 (Fig. 10E) levels and activated TGF-β1/Smad signaling pathway (Fig. 10F), but a reduced miR-342 expression (Fig. 10D) in liver tissues. NEAT1-depleted exosomes significantly suppressed the CCL_4_-induced effects on METTL3, NEAT1, miR-342, and Sp1 expressions and deactivated the TGF-β1/Smad signaling pathway. Nevertheless, treatment with NEAT1 overexpressed exosomes displayed the opposite functions (Fig. 10D–F). In summary, silence of NEAT1 in macrophage exosomes attenuated HF progression induced by CCL_4_ in vivo.

## Discussion

Previous studies demonstrated the continuous crosstalk between macrophages and HSCs [16]. Here, we for the first time demonstrated macrophage exosomal NEAT1 contributed to HSCs activation by sponging miR-342 and thus induced its downstream Sp1/TGF-β1/Smad signaling pathway in HF. Thus, NEAT1-depleting macrophage exosomes and miR-342 may potentially be developed as new therapeutic targets for HF treatment.

NEAT1 played a crucial role in mediating inflammatory response in macrophages. For example, Li et al. showed that NEAT1 increased LPS-induced inflammatory response in macrophages [17]. METTL3 was suggested to drive pro-inflammatory M1 macrophage polarization [4]. Though both METTL3 and NEAT1 were involved in mediating inflammation, the relationship between METTL3 and NEAT1 was not documented. To the best of our knowledge, we proved for the first time that METTL3 directly bound to NEAT1. Upon LPS treatment, the METTL3 level was increased in THP-1 macrophages and resulted in enhanced NEAT1 expression. We also firstly proved that NEAT1 was highly expressed in exosomes derived from LPS-induced THP-1 macrophages and primary Kupffer cells, and exosomal NEAT1 promoted HSCs proliferation and migration as well as fibrogenesis of HSCs. Also, consistent with in vitro data, NEAT1 overexpressed macrophage exosomes promoted HF in vivo. To the best of our knowledge, this study first time indicated the impact of macrophage exosomal NEAT1 on liver fibrosis.

We further validated that NEAT1 could directly bind to miR-342 through dual-luciferase assay and RIP assay. MiR-342 was indicated as a tumor suppressor in hepatocellular carcinoma [12, 18]. It was also documented by Jiang et al. that miR-342 suppressed renal interstitial fibrosis as evidenced by the decreased fibrosis markers [11]. However, the roles of miR-342 in HF were not reported before. Here, we showed that knockdown of miR-342 reversed the stimulation effects of NEAT1 overexpressed exosomes on HSCs proliferation, migration, and fibrosis-associated markers. These data further confirmed that exosomal NEAT1 regulated HSCs activation by sponging miR-342. As far as we known, this is the first time that reported the regulatory effects of miR-342 on liver fibrosis.

Qu et al. showed that suppressing TGF-β1-induced Smad2/Smad3 phosphorylation attenuated fibrosis [15]. TGF-β1 can be activated by the transcriptional factor Sp1 [19]. Here, we proved that Sp1 was a direct target of miR-342. In addition, Sp1 overexpression rescued the inhibitory effects of miR-342 overexpression on HSCs proliferation, migration and fibrosis-related proteins. To our knowledge, this study for the first time investigated the interaction of miR-342 and Sp1.

In conclusion, our study showed that exosomal NEAT1 from macrophages promoted HSCs activation and induced the expression of fibrotic markers. Furthermore, we demonstrated the underlying network of NEAT1/miR-342/Sp1 axis via the TGF-β1/Smad signaling pathway, revealing a novel theoretical basis for the application of exosomal NEAT1 in HF treatment. Future studies will focus on determining whether other molecules such as circRNAs, miRNAs, or proteins presented in macrophage-derived exosomes could mediate HSCs activation and HF progression.

## Materials and methods

### Cell culture

The human monocytic leukemia cell line THP-1 was purchased from the American Type Culture Collection (ATCC, Manassas, VA, USA). The human HSCs LX-2 and 293T cells were obtained from the Cell Bank of the Chinese Academy of Sciences (Shanghai, China). THP-1 cells were maintained in RPMI 1640 medium (Gibco) supplemented with 10% fetal bovine serum (FBS, Gibco) and 1% penicillin-streptomycin (Gibco). THP-1 cells were incubated for 24 h at 37°C with PMA (25 ng/mL, Sigma-Aldrich) to differentiate into macrophages. THP-1-derived macrophages were further treated with 10 ng/mL LPS for 12 h. The LX-2 and 293T cells were incubated in DMEM (Gibco) supplemented with 10% FBS and 1% penicillin-streptomycin. All the cell lines included in this study have been authenticated by STR profiling and tested for mycoplasma contamination.

### Isolation of primary Kupffer cells and HSCs

Primary Kupffer cells and HSCs from mouse liver were isolated based on a previously reported protocol [20]. All procedures were approved by the Animal Care and Use Committee of the Second Xiangya Hospital of Central South University (Changsha, Hunan, China). The used 8-week-old male BALB/c mice were obtained from Shanghai SLAC Laboratory Animal Center (Shanghai, China).

### Cell treatments

LX-2 cells were seeded into six-well plates. Cells were treated with either 50 µg of exosomes from THP-1 cells diluted in 2 mL DMEM medium or 2 mL conditioned medium (CM) from LPS-activated THP-1 cells or CM from LPS-activated THP-1 cells treated with GW4869 (Sigma-Aldrich), which was well accepted to inhibit exosome generation [21], before performing further experiments. Primary HSCs isolated from mouse liver tissues were also seeded into six-well plates, and treated with 50 µg of exosomes from LPS-activated Kupffer cells. Cells were also transfected with lentivirus pLCDH vectors (GeneCopoeia) harboring NEAT1, shNEAT1, METTL3, or shMETTL3 to overexpress (OE) or silence NEAT1/METTL3 expression. Lentiviral and packaging vectors were co-transfected into 293T cells to generate lentivirus. 48 h after the transfection, the supernatant was collected and concentrated using Lenti-Pac Lentivirus Concentration Solution (GeneCopoeia) according to the manufacturer’s instruction. After determining the titer, lentivirus was transduced into target cells at a multiplicity of infection (MOI) of 50 in the presence of polybrene (8 µg/mL, Sigma-Aldrich) overnight. shSp1, pcDNA-3.1-Sp1, miR-342 mimics, miR-342 inhibitor or their related negative controls (GenePharma) were also transfected into cells for 48 h using Lipofectamine 2000 (Invitrogen) without serum following the manufacturer’s instruction.

### Exosome isolation and characterization

To isolate the exosomes from the cells, total exosome isolation reagent (Cat# 4478359, Thermo Fisher Scientific) was used based on a previously published study [2]. In brief, THP-1 macrophages or Kupffer cells were cultured in culture plates until they reached 70% confluence. Then, cells were washed with phosphate-buffered saline (PBS, Gibco) and further incubated with 10% exosome-depleted FBS (Gibco) for 24 h. Afterward, the cell culture medium was collected and then was centrifuged at 2000 × *g* for 30 min, and the supernatant was filtered through a 0.22 mm filter (Sigma-Aldrich). The exosome isolation reagent (Invitrogen) was added to the medium and incubated overnight at 4 °C, following with centrifugation at 10,000 × *g* at 4 °C for 1 h. The exosome pellets were resuspended in 150 μL PBS and measured using NTA and TEM.

### Exosome uptake experiment

The exosomes from macrophages were marked by the PKH67 Fluorescent Cell Linker Mini Kit (Sigma-Aldrich) according to its manufactory’s instruction. The exosomes marked by PKH67 and HSCs were co-cultured for 12 h and subsequently fixed by 4% paraformaldehyde and stained by 2,4-diamino-5-phenylthiazole (DAPI, Sigma-Aldrich). The fluorescent images were taken with a Zeiss LSM 800 laser scanning confocal microscope (Zeiss, Germany). The measuring process was conducted by an assessor blind to treatment allocation.

### Dual-luciferase reporter assay

The assay was performed based on a previously reported protocol [22]. In brief, the NEAT1 wild-type sequence (NEAT1-WT) or Sp1 wild-type sequence (Sp1-WT) containing the binding site of METTL3 or miR-342 and the NEAT1 mutant sequence (NEAT1-MUT) or Sp1 mutant sequence (Sp1-MUT) were amplified and subsequently cloned into a psiCHECK2 vector (Promega). Afterward, the LX-2 cells were co-transfected with NEAT1-WT, NEAT1-MUT, Sp1-WT or Sp1-MUT and miR-342 mimics or miR-342 inhibitor and shMETTL3 or pcDNA-3.1-METTL3, utilizing Lipofectamine 2000 transfection reagent (Invitrogen). The Dual-Luciferase Reporter assay Kit (Promega) was used to examine the luciferase activities following 48 h incubation.

### RNA immunoprecipitation (RIP) assay and m6A RNA RIP assay

THP-1 cells, Kupffer cells, or LX-2 cells were collected, and RIP assay was performed using EZ-Magna RIP RNA-Binding Protein Immunoprecipitation Kit (Millipore) to identify the interaction of METTL3, NEAT1 and miR-342. In brief, RIP lysis buffer was added to the cells that were incubated with magnetic beads conjugated to either human anti-METTL3 antibody (1:50, ab195352, Abcam), human anti-Ago2 antibody (1:50, ab186733, Abcam) or the nonspecific anti-IgG antibody (1:50, ab172730, Abcam) at 4 °C overnight. Afterward, protein K buffer was added for another 2 h at 4 °C. After centrifuging five times, the co-immunoprecipitated RNA was isolated and used for further qRT-PCR analysis.

m6A RIP assay was performed utilizing the Magna m6A RNA PIP Kit (Millipore) based on the manufacturer’s instruction. In brief, THP-1 cells that were transfected with shMETTL3 or shNC were incubated in lysis buffer, then incubated with anti-m6A antibody (1:100, ab208577, Abcam), which was conjugated with protein A/G magnetic beads at 4 °C for 12 h. After centrifuging five times, the m6A-modified RNA was enriched and subjected to qRT-PCR analysis.

### m6A RNA methylation assay

The total RNA was isolated from THP-1 cells and the level of m6A RNA was measured with m6A RNA methylation Assay Kit (Abcam) according to the manual’s instruction.

### Cell proliferation detection by Cell counting kit-8 (CCK-8) assay

The proliferation of LX-2 cells and primary HSCs was assessed utilizing the CCK-8 assay (Dojindo, Japan). In brief, cells were seeded into 96-well plates with a density of 2000 cells/well. Following 12 h incubation to let the cells attach, 10 µL of CCK-8 solution was added into each well. Following 2 h incubation, the absorbance at 490 nm was obtained by a microplate reader (Thermo Fisher Scientific).

### Cell cycle assay

LX-2 cells were trypsinized and washed twice with PBS (Gibco). The cells were fixed with 70% ethanol. 24 h later, the cells were stained with propidium iodide (PI) by the CycleTEST PLUS DNA Reagent Kit (BD Biosciences) based on the manufacturer’s instructions. Subsequently, the cells were analyzed using the flow cytometer (Becton Dickinson, USA) and the cell cycle was analyzed by the Cell Quest Modfit software.

### Scratch assay

LX-2 cells and primary HSCs were seeded onto the 24-well plates until they reached a confluent monolayer. A 10 µL pipette tip (Eppendorf, Germany) was used to create a scratch. Afterward, the new cell culture medium without serum was replaced. Following 24 h of culture, the migrated distance was calculated under a light microscope (Zeiss, Germany). The measuring process was conducted by an assessor blind to treatment allocation.

### Transwell migration assay

LX-2 cells and primary HSCs were trypsinized, washed with PBS and were resuspended in a serum-free medium in a density of 5 × 10^5^ cells/mL. The cells were seeded into the upper chamber of transwell inserts (Millipore) with 8 µm pores. DMEM containing 10% FBS was added to the bottom chamber. 48 h later, the migrated cells were fixed in 4% paraformaldehyde, stained with 1% crystal violet. The stained cells were counted under the light microscope (Zeiss, Germany). The measuring process was conducted by an assessor blind to treatment allocation.

### Animal study

All experiments were approved by the Animal Care and Use Committee of the Second Xiangya Hospital of Central South University (Changsha, Hunan, China). Male Sprague-Dawley rats (6 week old, 180 ± 20 g) were obtained from Shanghai SLAC Laboratory Animal Center (Shanghai, China) and randomized into 6 groups (*n* = 10 per group): the control group, the CCL_4_ treatment model group, the CCL_4_ + OE-NC-Exo group, the CCL_4_ + OE-NEAT1-Exo group, the CCL_4_ + shNC-Exo group, and the CCL_4_ + shNEAT1-Exo group. For the model groups, 0.4 mL of CCL_4_/olive oil (3:2, v/v) per 100 g body weight was subcutaneously injected into rats in a frequency of 2 times per week for 5 weeks. For exosome treatment groups, the indicated exosomes were intravenously injected through the tail vein into rats twice a week for 5 weeks. Rats were sacrificed after 5 weeks, and the liver tissues and serum were collected for further experiments. The measuring process was conducted by an assessor blind to treatment allocation.

### Histological analysis

Liver tissues were fixed in 4% paraformaldehyde and were subsequently embedded in paraffin. Then the samples were sliced into 5 µm sections and were performed with histological analysis as previously reported [23]. Briefly, Masson staining of the sections was performed using a Masson’s Trichrome Stain Kit (Sigma-Aldrich) and imaged using a light microscope. Sirius Red staining was performed using a Picro Sirus Red Stain Kit (ab150681) from Abcam according to the manufacture’s instruction. In brief, sections were incubated in Picro Sirus Red solution for 60 min and followed by two times quickly rinsing in acetic acid solution and absolute alcohol. The images were also taken using a light microscope (Zeiss, Germany). The measuring process was conducted by an assessor blind to treatment allocation.

### Enzyme-linked immunosorbent assay (ELISA) assay

The ALT and AST levels in the rat serum were measured using ELISA kits (Abcam) based on the manufacturer’s protocol.

### Western blot

The total protein was isolated from cells or tissues using RIPA buffer (Abcam) supplemented with protease and phosphatase inhibitor. The total protein was measured using the BCA protein assay Kit (Thermo Fisher Scientific). 20 µg of protein was separated with SDS-PAGE and transferred onto nitrocellulose membranes (Millipore). Subsequently, the membranes were blocked with 5% skim milk and incubated with primary antibodies overnight at 4 °C. Afterward, the membranes were incubated with the corresponding anti-mouse or anti-rabbit IgG secondary antibodies for 2 h at 37 °C, and then the protein bands were visualized by ECL reagent (Sigma-Aldrich). The gray value was determined using Image J analysis software. All the antibodies used in this study were purchased from Abcam (CD63, ab216130; TSG101, ab125011; CD9, ab223052; collagen I, ab260043; α-SMA, ab5694; fibronection, ab2413; METTL3, ab240595; Sp1, ab227383; TGF-β1, ab215715; p-Smad2, ab280888; p-Smad3, ab63403; Smad2, ab33875; Smad3, ab40854; GAPDH, ab8245).

### Immunohistochemistry (IHC) staining

Liver tissues were fixed in 4% paraformaldehyde and were subsequently embedded in paraffin. IHC staining was performed on 5 μm slices and stained using the protocol as described previously [24]. In brief, the sections were deparaffinized in xylene and rehydrated in graded alcohols, followed by blocking the endogenous peroxidase activity in 0.3% hydrogen peroxide. The sections were then incubated with primary antibodies against collagen I (1:100, ab270993, Abcam), α-SMA (1:200, ab124964, Abcam), METTL3 (1:100, ab195352, Abcam), and Sp1 (1:500, SAB4502837, Sigma-Aldrich) at 4 °C overnight, followed by the incubation with the biotinylated anti-rabbit secondary antibody (1:1000, ab6720, Abcam) for 1 h at room temperature. Afterward, the avidin peroxidase conjugate (1:2000, ab59653, Abcam) was added for 1 h at room temperature. Finally, the target signal was developed utilizing DAB substrate (Vector Labs), and the slices were stained with hematoxylin. The images were taken under a light microscope (Zeiss, Germany). The measuring process was conducted by an assessor blind to treatment allocation.

### Real-time polymerase chain reaction (qRT-PCR)

The total RNA was extracted from cells or tissues using the Trizol reagent (Beyotime). 2 µg of total RNA was reversely transcribed using TaqMan^®^ microRNA reverse transcription Kit (Thermo Fisher Scientific) or PrimeScript RT Kit with gDNA Eraser (Takara) based on the manufacturer’s instructions. qPCR was carried out utilizing Taqman^®^ microRNA assay Kit (Thermo Fisher Scientific) or Taqman^®^ Universal PCR mixture Kit (Thermo Fisher Scientific) based on the manufacturer’s manual. The relative mRNA levels were quantified using the 2^−△△Ct^ method and normalized to GPDAH or U6. Primer sequences were purchased from Sangon Biotech (Shanghai).

### Statistical analysis

All data were analyzed by GraphPad Prism 6.0 and presented as the mean ± standard deviation (SD). All the data meet the assumption of normal distribution. All experiments were performed in at least three biological replicates, and each biological replicate contained three technical replicates. Statistical differences were performed using unpaired two-tailed *t* test for comparison between two groups and one-way analysis of variance followed by Tukey post hoc test for multiple comparisons. *P* < 0.05 was considered statistically significant.

## Supplementary information

Original Data File

Supplementary materials

Supplementary fig.1

Supplementary fig.2

Author Contribution Form

## Data Availability

All data generated or analyzed during this study are included in this article. The datasets used and/or analyzed during the current study are available from the corresponding author on reasonable request.

## Material availability

Supplementary figure legends and original western blots were shown in Supplemental Materials.
