# Supplementary material for: METTL3-mediated macrophage exosomal NEAT1 contributes to hepatic fibrosis progression through Sp1/TGF-β1/Smad signaling pathway
Source: Cell Death Discov. 2022 May 19;8:266. doi: 10.1038/s41420-022-01036-y (PMC9117676; doi:10.1038/s41420-022-01036-y)
Supplement: Supplementary file 2 — Supplementary materials [file 41420_2022_1036_MOESM2_ESM.docx]

**Supplementary Figure Legends**

**Supplementary Figure 1. METTL3 promotes NEAT1 expression in Kupffer cells and NEAT1 expression is also increased in exosomes derived from LPS-treated Kupffer cells.**

(A-B) The METTL3 mRNA and protein levels were determined by qRT-PCR (A) and western blot (B) in METTL3 overexpressed or depleted Kupffer cells. (C) The NEAT1 expression level was determined by qRT-PCR in METTL3 overexpressed or depleted Kupffer cells. (D) METTL3 RIP-qPCR analysis of NEAT1 enrichment in Kupffer cells. (E-F) Characterization of exosomes isolated from Kupffer cells that isolated from mouse livers using TEM (E) and NTA (F) analyses. (G) Western blot analysis of the expression levels of CD63, TSG101 and CD9 in exosomes isolated from Kupffer cells. (H) Relative NEAT1 expression level in exosomes secreted from Kupffer cells treated with or without LPS. **p*<0.05, ***p*<0.01 and ****p*<0.001.

**Supplementary Figure 2. Exosomes extracted from LPS-treated Kupffer cells enhance the activation of primary HSCs.**

(A) Relative NEAT1 expression level in primary HSCs that treated with exosomes excreted from LPS-activated Kupffer cells or untreated Kupffer cells. (B) The proliferation of the primary HSCs was examined by the CCK-8 assay. (C-D) The migration of primary HSCs was determined by the scratch assay (C) and the transwell assay (D). (E) Western blot analysis of the protein levels of collagen I, α-SMA, fibronectin, Sp1, TGF-β1, p-Smad2, Smad2, p-Smad3 and Smad3 in primary HSCs. (F) Relative miR-342 expression in primary HSCs that treated with exosomes excreted from LPS-activated Kupffer cells or untreated Kupffer cells. **p*<0.05, ***p*<0.01 and ****p*<0.001.
