# Supplementary figures and images for: METTL3-mediated macrophage exosomal NEAT1 contributes to hepatic fibrosis progression through Sp1/TGF-β1/Smad signaling pathway
Source: Cell Death Discov. 2022 May 19;8:266. doi: 10.1038/s41420-022-01036-y (PMC9117676; doi:10.1038/s41420-022-01036-y)

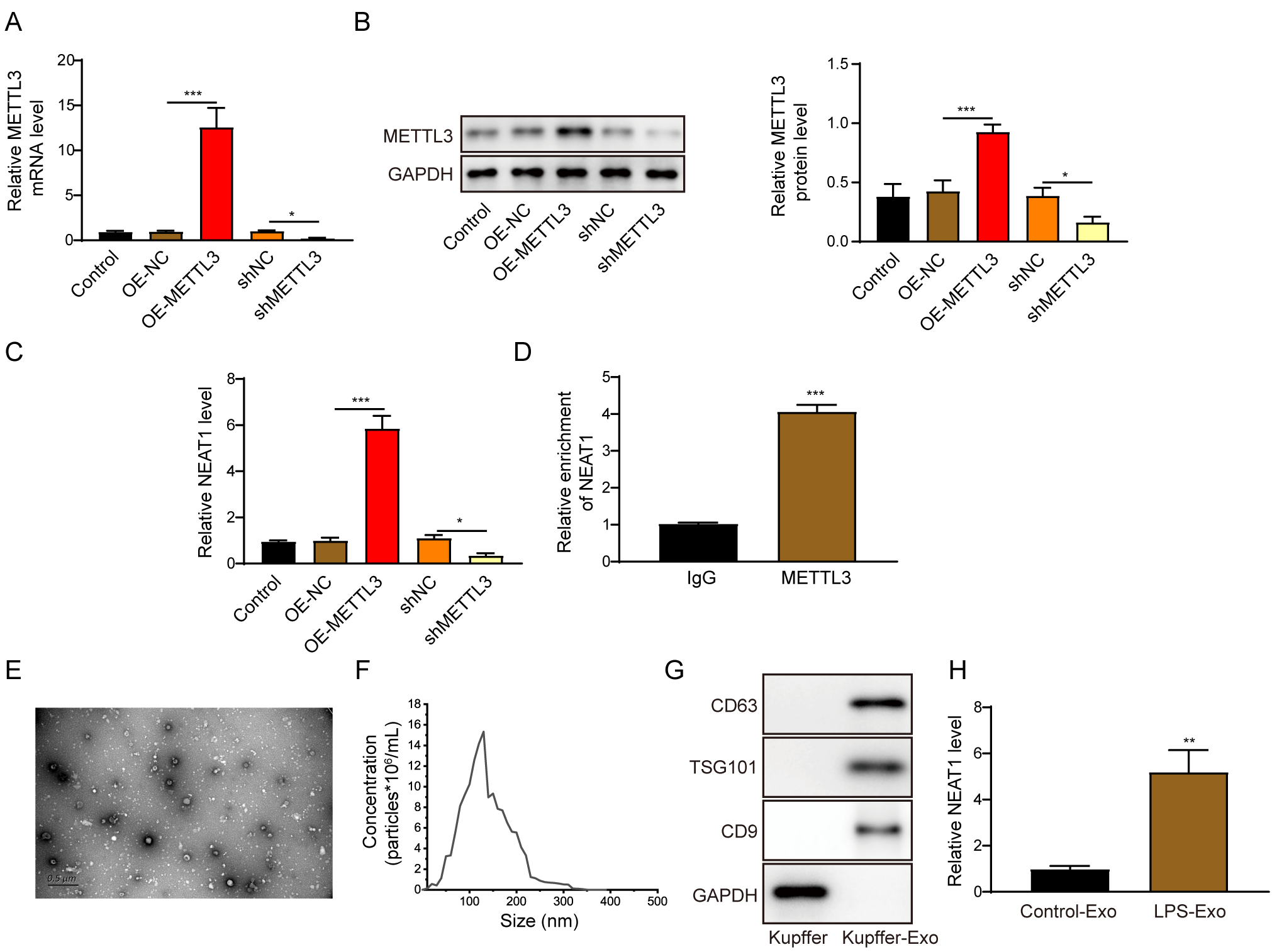

Supplement: Supplementary file 3 — Supplementary fig.1 [file 41420_2022_1036_MOESM3_ESM.tif]

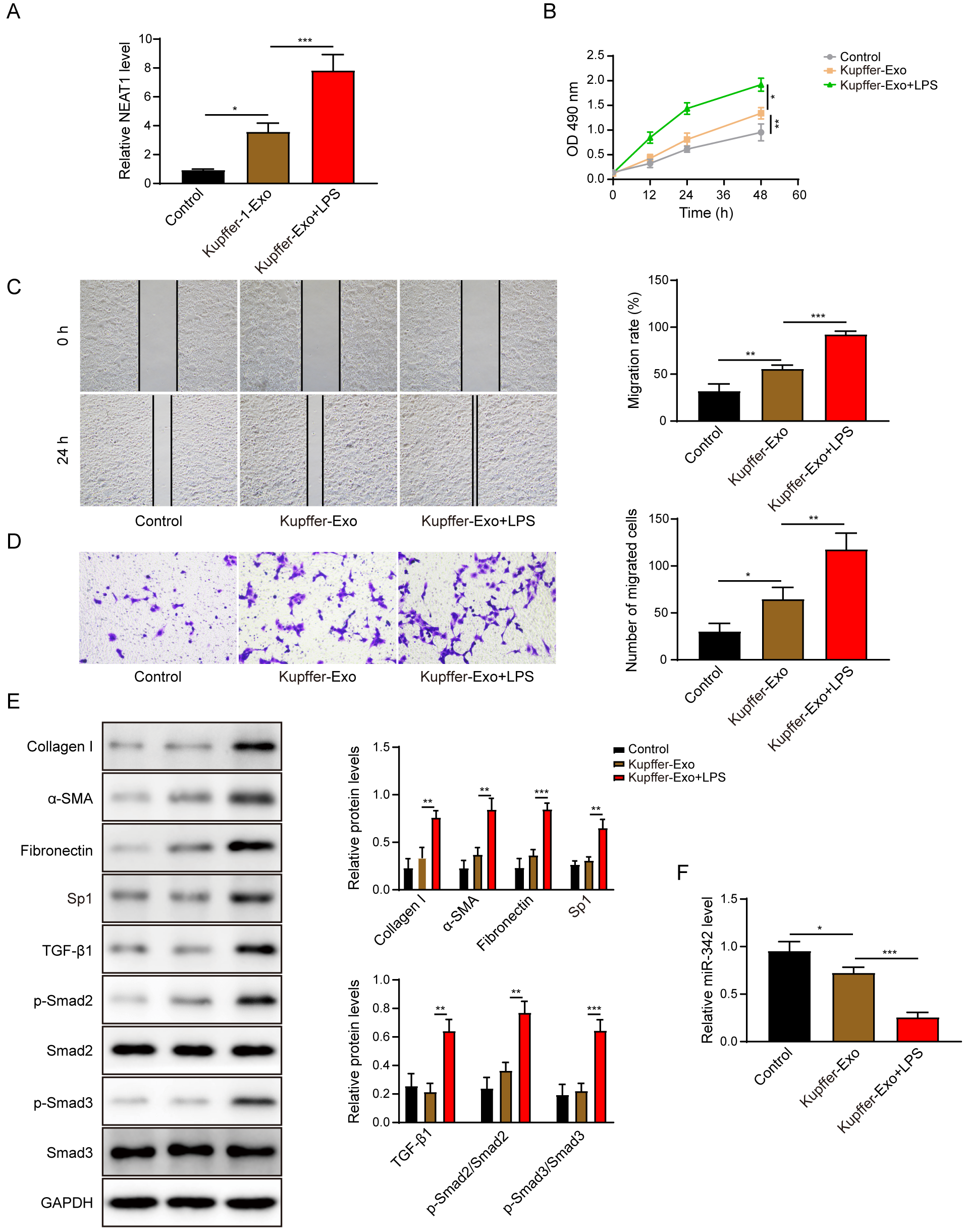

Supplement: Supplementary file 4 — Supplementary fig.2 [file 41420_2022_1036_MOESM4_ESM.tif]

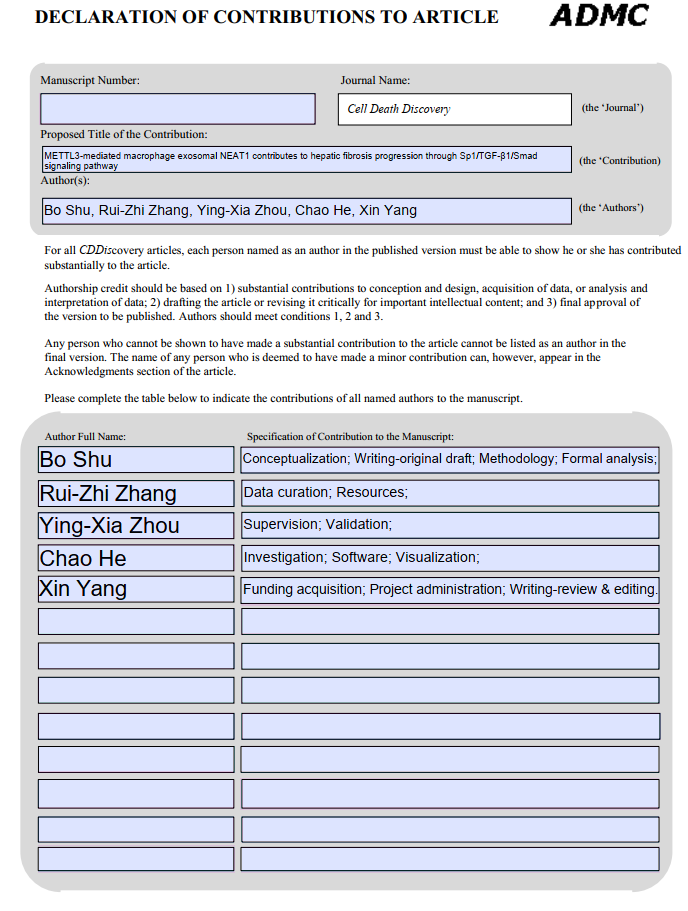


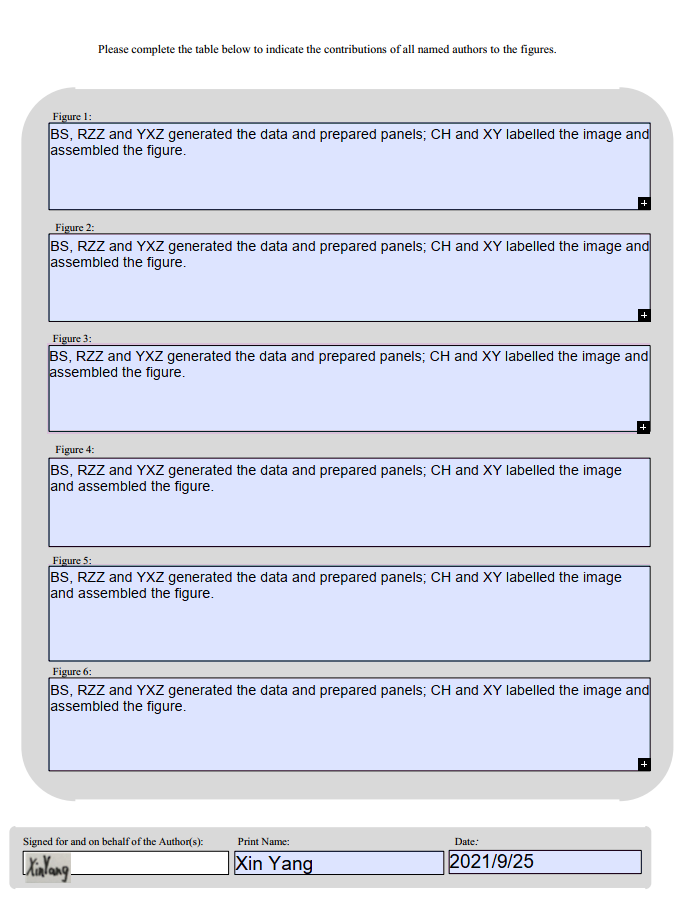

Supplement: Supplementary file 5 — Author Contribution Form [file 41420_2022_1036_MOESM5_ESM.docx]
